# Child Health and Mortality Prevention Surveillance (CHAMPS): Manhiça site description, Mozambique

**DOI:** 10.12688/gatesopenres.13931.2

**Published:** 2024-09-05

**Authors:** Charfudin Sacoor, Pio Vitorino, Ariel Nhacolo, Khátia Munguambe, Rita Mabunda, Marcelino Garrine, Edgar Jamisse, Amílcar Magaço, Elísio Xerinda, António Sitoe, Fabíola Fernandes, Carla Carrilho, Maria Maixenchs, Percina Chirinda, Tacilta Nhampossa, Bento Nhancale, Natalia Rakislova, Justina Bramugy, Arsénio Nhacolo, Sara Ajanovic, Marta Valente, Arsénia Massinga, Rosauro Varo, Clara Menéndez, Jaume Ordi, Inácio Mandomando, Quique Bassat

**Affiliations:** 1 Manhiça Health Research Center, Municipio da Vila da Manhiça, Maputo Province, 1929, Mozambique; 2 Faculty of Medicine, Eduardo Mondlane University, Maputo, Maputo, Mozambique; 3 Department of Pathology, Maputo Central Hospital, Maputo, Maputo, Mozambique; 4 ISGlobal, Hospital Clinic, Universitat de Barcelona, Barcelona, Spain; 5 Department of Pathology, Hospital Clinic, Universitat de Barcelona, Barcelona, Spain; 6 CIBER Epidemiologia y Salud Publica (CIBERESP), Barcelona, Spain; 7 National Institute of Health, Ministry of Health of Mozambique, Maputo, Mozambique; 8 ICREA, Barcelona, Spain; 9 Pediatric Department, Hospital Sant Joan de Deu- Universitat de Barcelona, Barcelona, Spain

**Keywords:** Manhiça site description, Health and Demographic Surveillance System, morbidity surveillance, Mozambique, child mortality, post-mortem, MITS, CISM.

## Abstract

The Manhiça Health Research Centre (Manhiça HDSS) was established in 1996 in Manhiça, a rural district at Maputo Province in the southern part of Mozambique with approximately 49,000 inhabited households, a total population of 209.000 individuals, and an annual estimated birth cohort of about 5000 babies. Since 2016, Manhiça HDSS is implementing the Child Health and Mortality Prevention Surveillance (CHAMPS) program aiming to investigate causes of death (CoD) in stillbirths and children under the age of 5 years using an innovative post-mortem technique known as Minimally Invasive Tissue sampling (MITS), comprehensive pathogen screening using molecular methods, clinical record abstraction and verbal autopsy. Both in-hospital and community pediatric deaths are investigated using MITS. For this, community-wide socio-demographic approaches (notification of community deaths by key informants, formative research involving several segments of the community, availability of free phone lines for notification of medical emergencies and deaths, etc.) are conducted alongside to foster community awareness, involvement and adherence as well as to compute mortality estimates and collect relevant information of health and mortality determinants. The main objective of this paper is to describe the Manhiça Health and Demographic Surveillance System (HDSS) site and the CHAMPS research environment in place including the local capacities among its reference hospital, laboratories, data center and other relevant areas involved in this ambitious surveillance and research project, whose ultimate aim is to improve child survival through public health actions derived from credible estimates and understanding of the major causes of childhood mortality in Mozambique.

## Disclaimer

The views expressed in this article are those of the author(s). Publication in Gates Open Research does not imply endorsement by the Gates Foundation.

## Introduction

The Child Health and Mortality Prevention Surveillance ( CHAMPS) program was established in 2015 as a multisite network whose primary aim is to conduct comprehensive child mortality surveillance in sub-Saharan Africa and South Asia. CHAMPS uses innovative post-mortem and community-wide demographic approaches, as a long-term strategy to better understand the causes of death and to positively impact child survival ^1^. With an ambitious horizon of a 25-year lifespan, this network was conceived as a long-term endeavor to generate state-of-the-art data on child mortality in settings with high mortality burdens. Now in its 10 ^th^ year of activities, the network includes sites in six African countries (Mozambique, South Africa, Mali, Kenya, Ethiopia, and Sierra Leone) and one in Asia (Bangladesh), with plans of opening two other sites (Pakistan and Nigeria) during the year 2023.

The Manhiça Health Research Centre ( *Centro de Investigação em Saúde de Manhiça*; CISM), in Manhiça district, Southern Mozambique, was the pioneer site of CHAMPS activities within the network in the year 2016. The sharp innovation brought by CHAMPS in Manhiça was the inclusion, as a routine activity, of the minimally invasive tissue sampling technique [MITS, also known as Minimally Invasive Autopsy (MIA)] as part of the tool-kits used to investigate causes of death (CoD) in stillbirths and children under the age of 5 years ^2, 3^. This technique was validated for all age groups as an acceptable proxy to the complete diagnostic autopsy ^4, 5^, and due to its non-disfiguring nature, was found to be much more acceptable by family members of the deceased, permitting post-mortem sampling and bypassing the unsurmountable hurdles of conducting full pathological autopsies in African and Asian settings ^6– 8^.

Although the CHAMPS project is being implemented in two sites (Manhiça district and Quelimane district) this paper aims to describe the capacities of the Manhiça site and particularities of the study implementation in this district.

## Why CHAMPS in Mozambique?

CHAMPS is targeting high mortality burden countries (defined as those with a child mortality rate above 50/1000 live births) that already had the infrastructure to adequately track childhood mortality ^9^. Any site applying to become part of the network needed to have a well-established population under demographic surveillance, a birth cohort of 3,000 or more newborns per year, good clinical facilities and community liaisons within the study area, and reasonable coverage of antenatal consultations among pregnant women. Despite CISM providing robust data on mortality in Mozambique, including death registration and determination of cause of death through verbal autopsy, CHAMPS represents an opportunity to enhance this already robust infrastructure. In Manhiça, where over 50% of deaths occur at home, verbal autopsies (VA) within the HDSS are used to determine causes of death. However, verbal autopsies have limitations, including potential recall bias and variability in interpretations, which may affect the accuracy of cause-of-death determinations. Furthermore, death certificates, which are often incomplete or inaccurately completed, pose potential limitations even for facility deaths in accurately documenting causes of death, particularly in resource-constrained settings. CISM, a research center of excellence within the continent, fulfilled all these prerequisites and already had solid experience engaging with the community regarding the potential use of post-mortem techniques ^6^ and a long history of conducting verbal autopsies (VA) for mortality surveillance ^10^. In the following paragraphs, this paper describes the particularities of Mozambique as a country and Manhiça district as the chosen site to implement the first years of CHAMPS activities in the country. Additionally, it discusses the epidemiological profile and knowledge gaps regarding the main causes of childhood mortality in Manhiça, justifying its suitability to join the CHAMPS network.

## General country and Manhiça district characteristics

Mozambique is located on the southeastern coast of Africa, with an Indian Ocean coastline of 2,700 Km ^11^, bordering Tanzania in the North, Malawi and Zambia in the northwest, Zimbabwe in the west, and Swaziland and South Africa in the South. It covers 799,380 Km ^2^ and is administratively divided into 11 provinces which are commonly grouped into three geographical regions (the Northern, Central, and Southern regions) ^12^.

The most recent national census (2017) indicates that the country has 27.9 million inhabitants, of which 46% are children under the age of 15 years, and less than 3.3% are older than 65. The official language is Portuguese, but the people also speak Bantu languages. The country has high religious diversity, with about 60% of the population adhering to some form of Christianity or evangelism, followed by Muslims, who account for 19% of the population. Most of the Mozambican economy is based on primary sector activities such as agriculture, livestock, fishing and informal mineral extraction ^13^. The national currency is the Metical (1 USD ~ 63.29 MZN; according to the national Bank of Mozambique). The average annual inflation in Mozambique reached 5.7% in 2021, almost double the rate registered in 2020, and the GDP growth rate was 2.22%, a 3.46% increase from 2020 ^13^.

**Figure 1.**
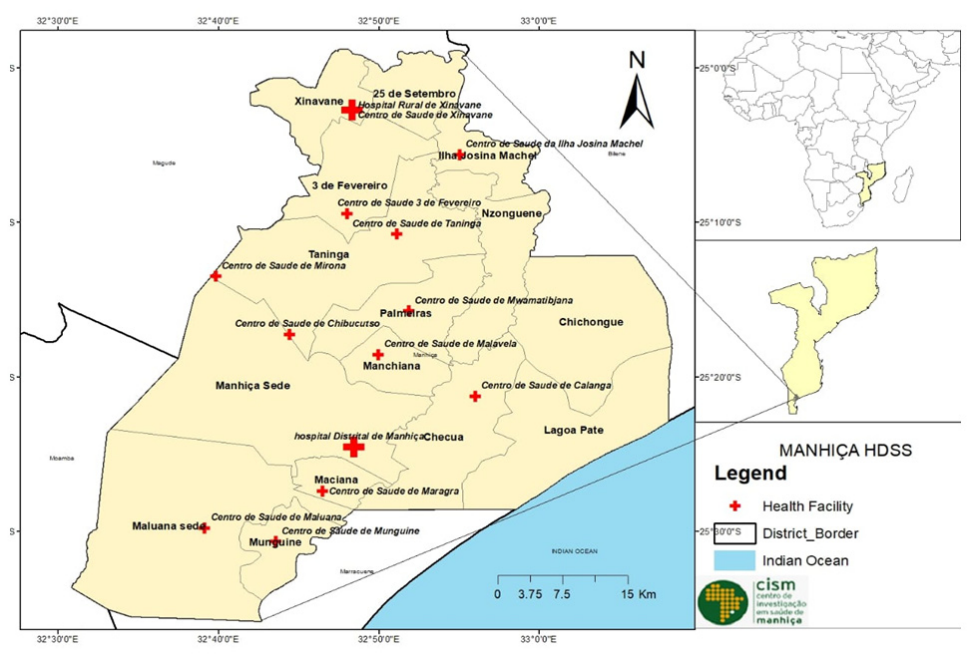
Map of Manhiça district (2021) covering the Manhiça Health and Demographic Surveillance System (HDSS), by the authors. (A) Administrative shape files were obtained from CENACARTA. (B) GPS positions of health facilities were obtained directly from the field by the authors.

The district of Manhiça is located about 90 km north of Maputo city, the capital of Mozambique. It has geographical limits, to the north and northeast with the district of Bilene Macia in the province of Gaza, to the east with the Indian Ocean, to the south with the district of Marracuene, to the west with the district of Moamba and to the west and northeast with the district of Magude. Figure 1 presents a map showing the location of Mozambique in Africa, and the Manhiça district including administrative divisions and health facilities.

The district is located on a plain, surrounded by the Incomati River, covering an area of 2.380km ^2^; This district has two distinct climate seasons. The warm season (November to April) coincides with most of the rainfall (900 – 1100 mm on average) and is followed by a cool and dry seasonover lasting for the rest of the year. The residents are mainly Xichangana and Xironga. The three predominant religions are Christian related Zion, Protestants and other Indeterminate Christianity. About 76% of the households were constructed by conventional materials in the district. 54% of the houses have electricity, 93% have conventional latrines and about 34% of houses have potable water within the compound. The majority of the study population are engaged in small businesses, subsistence farming, laborers in sugar cane plantations and sugar refining companies and other small agriculture companies ^13^.

**Figure 2.**
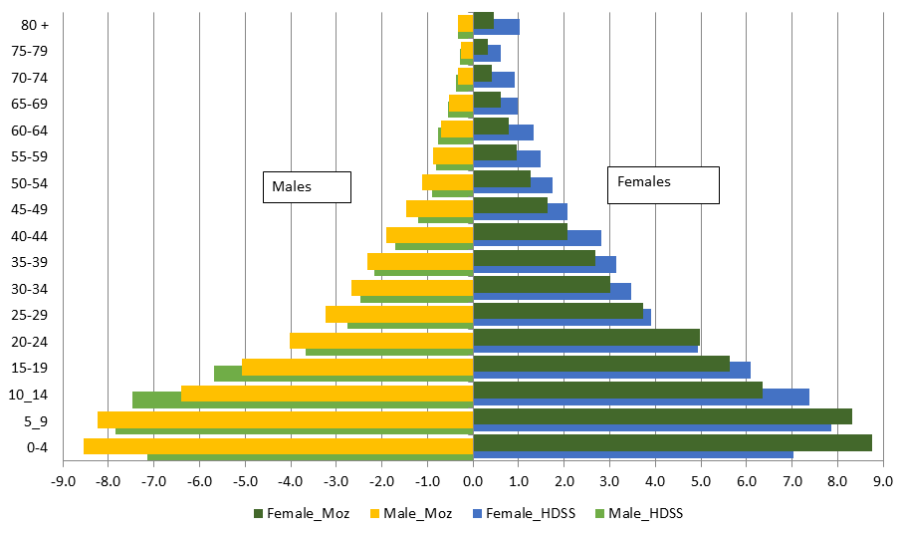
The population pyramid of Mozambique (Moz) in 2017 and the Manhiça district Health and Demographic Surveillance System (HDSS) in 2020. The X axis is the percent of the population for males (left graphs) and females (right graphs), and the Y axis is the age group in years. Female_Moz = % of females of this age group among the total national population; Male_Moz = % of males of this age group among the total national population; Female_HDSS – % of females of this age group among the total Manhiça HDSS population; Male_HDSS – % of males of this age group among the total Manhiça HDSS population; Note: negative % in males were made for graphical purposes only.

Figure 2 shows the age and sex composition of the population of Mozambique and Manhiça and suggests an expansive population pyramid, with a large base due to a high fertility rate and a narrower top due to high mortality rates, typical of most sub-Saharan Africa populations with lower life expectancy. Despite having a very young population, Mozambique – and Manhiça in particular – also face a huge burden of neonatal and under-5 mortality. This corresponds to a country that has not yet experienced its demographic transition and where infectious diseases still represent the primary cause of premature and preventable deaths. The epidemiological profile of Mozambique is characterized by infectious diseases such as HIV/AIDS, tuberculosis, malaria, lower respiratory infections and diarrhea, although non-communicable diseases such as stroke, ischemic heart diseases and road injuries are also important causes of death ^13^. In children, pneumonia, malaria, neonatal disorders, diarrheal disease, and malnutrition are among the most important causes of morbidity and mortality ^14, 15^.

Although the government efforts after the civil war in 1992 have improved the health services, medical care in Mozambique is still limited, with 8.7 medical doctors per 100,000 inhabitants and 0.74 beds per 1000 inhabitants ^16^. The National Health System is divided into 4 levels of health care units: (i) Primary Health Care Units (rural health centers type I and II, urban health centers type A, B, C with or without maternity hospital), (ii) Secondary Health Care Units (rural, district and general hospitals), (iii) Tertiary Health Care Units (provincial hospitals) and (iv) Quaternary Health Care Units (central and specialized hospitals). In total the country has 1,625 health facilities divided into four levels ^17^. There are only four hospitals of level four in the country, based in Maputo (South), Beira (Central), Quelimane (Central), and Nampula (North). The majority of the population of the country is served by health facilities of the lowest level; access to specialized health care services is limited.

Current national estimates of mortality indicate still unacceptably high figures, with neonatal, infant and under 5 mortality rates estimated at 30, 67, and 78 per 1000 live births, respectively, and a maternal mortality rate of 451,6 per 100,000 live births ^18– 20^. Table 1 presents a summary of selected demographic and health indicators in Mozambique and the Manhiça district.

**Table 1.** Selected demographic and health indicators in Mozambique and Manhiça HDSS.

Demographic and health indicators	Mozambique (year 2017)	Manhiça HDSS (year 2020)
Total population	27,909,798	207,339
Population density	35/km ^2^	87/km ^2^
Percentage of population that is urban	33.4%	0%
Percentage of population that is rural	66.6%	100%
Administrative divisions	11 provinces	6 administrative posts
Male adult literacy rate	72.8%	47.5%
Female adult literacy rate	50.6%	37.5%
Birth rate	38/1000	26.2/1000
Fertility rate, total (births per woman)	5	3
Neonatal mortality rate (per 1,000 live births)	30.1	13.6
Infant mortality rate (per 1,000 live births)	67.3	21.2
Mortality rate, under-5 (per 1,000 live births)	77.9	31.3

Source: *Instituto Nacional de Estatística* (INE) data from census carried out in 2017, UNICEF estimates of 2017 and CISM demographic data of 2020.

## The Manhiça Health and Research Center

The CISM was established in the Manhiça district in 1996 to conduct biomedical research on diseases affecting the poorest and most vulnerable populations. A full description of this center has been presented elsewhere ^21^ and recently updated ^22^. Manhiça is the paradigm of a poor, resource-constrained rural sub-Saharan African setting with a predominantly young population (18% <5 years of age) ^21^. The three pillars of the CISM activities include research, clinical assistance, and training of young investigators. Research activities are conducted under the umbrella of three well-established surveillance platforms, focusing on demography, morbidity, and microbiology, respectively. Over the past 25 years, the CISM has conducted a series of studies with an important impact on public health policies in the country, including studies on malaria preventive tools (RTS,S), a malaria candidate vaccine ^23^, intermittent preventive treatment in infants and intermittent preventive treatment in pregnancy (IPTi/IPTp) ^24, 25^), the treatment of malaria ^26, 27^, and the detailed description of the burden and epidemiology of malaria, childhood bacterial infections (including pneumonia or meningitis), moderate-to-severe diarrhea and other life-threatening infections in children ^28– 33^.

**Figure 3.**
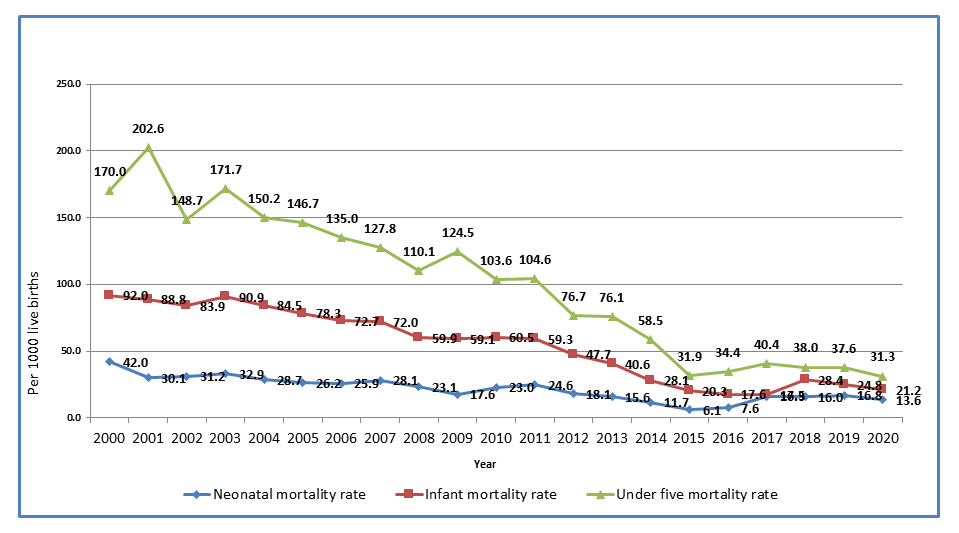
Trends of neonatal, infant and child mortality in Manhiça district from 2000 to 2020. Source: Manhiça HDSS databases, 2020.

Through the linkage of its three routine surveillance platforms, CISM has, in recent years, provided detailed descriptions of the community's health status, particularly its pediatric mortality and morbidity indicators. When the CHAMPS network was established in 2014, and Manhiça applied as a potential member, the under 5 mortality rate in the area was 58.5/1000 live births. Estimates of 2020 neonatal, infant and under-five mortality rates in Manhiça were 13.6/1000 live births 21.2/1000 and 31.3/1000, respectively, showing a steady decreasing trend in the past two decades ( Figure 3).

Additionally, the latest estimates of HIV prevalence in the community (39.9% seropositivity among adults) ^34^ confirm the remarkably high burden imposed by this infection, and in recent years, a cohort of around 4000 HIV-positive children has been routinely followed at the HIV outpatient consultation in Manhiça district hospital (MDH). CISM has conducted etiological surveillance for the most common infections affecting children and infants in the area, including malaria, pneumonia, and meningitis, among others ^35^.

## PRE- AND POST-CHAMPS CAPACITY IN CISM

### Health and Demographic Surveillance System (HDSS)

HDSS has been in place and running since the year 1996 in Manhiça, currently covering the totality of the population of the district. The HDSS includes a full population census that is regularly (twice a year) updated and a detailed registry of all major demographic events (births, deaths, pregnancies, in- and out-migrations). This platform supports the design and implementation of all the biomedical research activities conducted by the CISM either in Manhiça or elsewhere in the country, including verbal autopsy (VA). The HDSS currently comprises approximately 209,000 inhabitants living in approximately 48897 households (HDSS unpublished data of 2022). Manhiça and other rural districts in southern Mozambique are primary sources of migrants to South Africa, Eswatini, and Maputo. Historically, these areas provided many labor migrants for mines and farms, and to seek better conditions in the capital. In 2019, before the COVID-19 pandemic, the crude external out-migration rate was 85.8 per 1000 person-years (pyrs) for males and 77.7 per 1000 pyrs for females. In 2021, during the pandemic, these rates decreased to 65.3 per 1000 pyrs for males and 60.4 per 1000 pyrs for females, particularly among the 15-39 age group. For external in-migration, 2019 rates were 46.3 per 1000 pyrs for males and 43.7 per 1000 pyrs for females. In 2021, these rates dropped to 27.3 per 1000 pyrs for males and 27.5 per 1000 pyrs for females. There was also a notable in-migration of children under five, with rates of 40-68 per 1000 pyrs, likely brought to stay with grandparents or accompanying returning parents

The HDSS data have allowed the calculation of accurate estimates of vital events in different population sub-groups to measure disease incidence and a continuous surveillance of the main causes of death for all ages since the establishment of the platform. Data collection procedures have been described elsewhere ^21^. Briefly, it includes three types of visits, namely (i) yearly rounds that were routinely increased to every six months as a result of CHAMPS; (ii) weekly visits to key informants (about 170); and (iii) daily visits to the health facilities to gather information on pregnancies in prenatal consultations as well as deaths and pregnancy outcomes that have occurred in the past 24 hours.

Prior to CHAMPS, deaths had been captured mainly through: (i) routine home visits by field workers assigned to neighborhoods containing a a number of households that could each be visited over 5 months, targeting at least 10 households per day; (ii) daily visits to health facilities by supervisors to gather information on facility deaths; (iii) home visits to key informants (such for instance community and religious leaders among others) by supervisors to collect events that occurred in their neighborhoods in the preceding week. All vital events, including deaths, are linked to the individual permanent identification Number (perm_id) assigned by the HDSS to each household member that meets HDSS inclusion criteria (voluntarily sign an informed consent and live or have an intention to live in one specific household for at least 3 months in Manhiça District) ^21, 36^. The Manhiça HDSS has the strength of providing accurate, ongoing, and periodically updated household and individual data, including all vital events and migration. It also collects essential socio-demographic information such as household characteristics and socio-economic status. This platform is crucial for understanding the impact of local contextual, environmental, demographic, and programmatic factors on health outcomes. In Manhiça, data on outpatient and inpatient visits of children under 15 years, including those under 5 years in the CHAMPS study, are systematically collected within the HDSS. Additionally, the HDSS facilitates clinical trials, monitors national and global health interventions and programs, and computes accurate demographic and epidemiological indicators. However, due to spatial limitations, the data generated within the HDSS cannot be generalized to larger populations. Cost and respondent fatigue are also notable weaknesses of this method.

However, as CHAMPS required the timely detection of under 5 deaths and stillbirths within 24 hours of occurrence to allow the performance of MITS without interference in the burial process, the HDSS established a call center (open 24h/24h, 365 days per year) to receive real-time notifications from key informants in the community, staff from health facilities, the police, and other community members – who were mobilized to report deaths and other demographic events as soon as they became aware of them. In the context of CHAMPS activities, the CISM provided mobile phones to the key informants who did not have them.

When a new death is notified, the call center registers the information in an existing database. It ensures that potential eligibility for CHAMPS post-mortem activities is assessed and that within the following 30 days, field workers visit the household in order to conduct the VA for that death. This is another major change given that, before the initiation of CHAMPS, VAs were typically conducted several months after the demise. The most important change, however, is the speed by which families are contacted to ascertain the feasibility of MITS within the pre-specified maximum hours (<36) after their child's death. The increase in the last years of community deaths that have been detected and in which MITS has been successfully conducted confirms that the system to identify deaths rapidly is adequately working.

Additionally, a significant change was made to the routine HDSS of the CISM was the assignment of Perm_ID numbers for stillbirths, a population group that was, before CHAMPS, counted but not routinely part of the surveillance. Including them as part of the HDSS allowed much closer surveillance and the possibility of conducting, as the CHAMPS protocol requires, a VA within 30 days to any stillborn detected in the district.

## Morbidity and mortality surveillance

### Morbidity surveillance

The morbidity surveillance system, ongoing since 1998 at MDH and five peripheral health facilities ^37^, helps document all pediatric out- and inpatients visits (using standardized forms that include demographic information, clinical history, clinical exam, outcome and treatments completed by a trained health professional) for all children <15 years of age attending these health units. Standardized data on over ~70,000 pediatric admissions and 1.45 million outpatient visits have been collected over the past two decades, making up one of the largest health databases in the country. Malaria screening (for all children with fever or a history of fever in the preceding 24 hours) and microbiological surveillance of invasive bacterial disease have been in place at MDH as a routine practice by performing systematic blood culture collection for all children <2 years of age that are admitted, and for older children with suspected severe disease, including cerebrospinal fluid for meningitis suspected cases. This platform-generated data are critical for defining and evaluating public health policies in Mozambique, such as the introduction of *Haemophilus influenzae* type b (2009), pneumococcal conjugate (2013) and rotavirus (2015) vaccines in the Mozambican immunization program ^29– 32, 38^. Introducing such vaccines was associated with significant drops in child mortality ( Figure 3).

The thorough and continuous morbidity surveillance established by CISM has allowed the running of complex epidemiological studies and clinical trials that require an adequate linkage between field activities, clinical characterization and outcomes. The clinical department of the CISM has played a pivotal role in establishing such links. It is also responsible for establishing the communication and coordination bridge between CISM and health authorities, ensuring that all activities carried out by CISM are under rules established by the National Health System, with particular emphasis on deploying and following nationally established protocols and other related activities.

## Surveillance of causes of death: from verbal autopsies to MITS

The CISM has been a pioneer in conducting mortality surveillance. As one of the founding members of INDEPTH, the global network of population surveillance in low- and middle-income countries ^39^, health and population dynamics have been systematically monitored over the past 24 years. Causes of death are captured using the standardized WHO verbal autopsy form. Current data collection is done using Open Data Kit ( ODK ^©^ ) based on the OpenHDS platform. An in-house electronic application was developed using a customized WHO 2016 VA, and has already been introduced as part of the routine surveillance. A retrospective analysis of VA data for children in the period 1997–2006 highlighted the importance of infections, particularly malaria, pneumonia and diarrheal diseases, as the main threats to child survival in the district ^10^. However, recognizing the limitations of data generated through VA ^40^, researchers at CISM wanted to promote the use of more reliable methods to investigate causes of death. A first study conducted in 2004 at the pathology department of Maputo Central Hospital (MCH), in which maternal deaths were investigated by full diagnostic autopsy, confirmed the remarkable role that infectious diseases played in terms of maternal mortality ^41^ and the high frequency of discrepancy between clinical diagnosis and pathology findings elucidated by post-mortem investigations ^42^. Such results highlighted the need for more robust approaches to investigate the causes of death, recognizing the significant barriers to acceptability that conventional post-mortem approaches may pose to communities. In this context, and again benefitting from research funding from the Bill and Melinda Gates Foundation, and a revolutionary Minimally invasive autopsy (MIA) approach was designed ^43, 44^. In this approach, tissue samples of the key organs together with bodily fluids (blood, CSF etc.) were obtained using fine biopsy needles, a method that left little or no marks on the body and was found to be acceptable, with consent rates approaching 70–80% ^3, 45^. The validation studies, also conducted in MCH, created the path to implementing this post-mortem approach in rural settings like Manhiça, where such a technique was innovative and could provide useful and actionable data. With the advent of CHAMPS, the implementation of MIA (subsequently termed MITS to avoid the use of "autopsy" in the term) became a reality. The first MITS conducted as part of the entire CHAMPS network was done on Dec 9 ^th^ 2016, in the Manhiça District Hospital (MDH). Since then, nearly 500 MITS have been conducted at either MDH or the neighboring Xinavane hospital, including deaths occurring at the community level.

**Figure 4.**
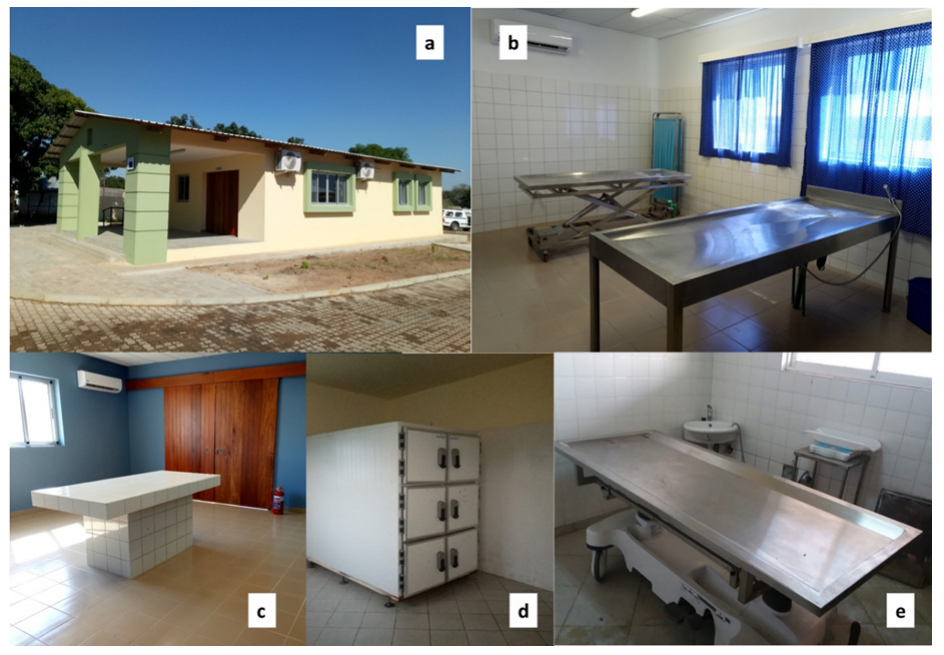
Renewed morgue ( **a**) and post-mortem facilities at Manhiça district hospital ( **b**, **c**). Refrigerator room ( **d**) and post-mortem table ( **e**) at the renewed morgue of Xinavane Rural Hospital, used for CoD investigation and mortality surveillance.

CHAMPS has contributed to improving clinical infrastructures in the Manhiça District, both at MDH and at the neighboring Xinavane Rural Hospital. In this setting, CHAMPS activities have allowed the surveillance and investigation of childhood deaths and stillbirths using MITS. Such improvements included renovation of morgue facilities in both settings ( Figure 4), allowing corpse refrigeration and post-mortem investigations using MITS. Such infrastructures were developed in collaboration with the pathology departments of the Hospital Clínic in Barcelona, Spain, and the Maputo Central Hospital, given their previous involvement in the post-mortem studies leading to the validation of the minimally invasive autopsy ^43^.

CHAMPS has also enhanced the existing collaboration between CISM and the District, Provincial and National health authorities, particularly for "data-to-action" activities, in which CHAMPS data are immediately shared at different levels and areas for improvement are identified for appropriate action. Data-to-action activities include any CHAMPS data-driven activities with the potential to improve child survival. As an example, CHAMPS surveillance strengthened the referral of critically ill patients from remote areas to the MDH and, when necessary, to the MCH. Hospital support in terms of medical supplies and surgical equipment for the health units where CHAMPS surveillance is in place has substantially contributed to the improvement of health care for patients, particularly regarding oxygen administration and enhanced provision of care at the maternity level. Furthermore, the expansion of morbidity surveillance as described above to Xinavane Rural Hospital will contribute to defining the burden of infectious diseases in this administrative post and help better guide local policies. CHAMPS has also contributed to continuous capacity building and training at all levels. For example, CHAMPS support has enabled clinical, laboratory and demography investigators to pursue – through CHAMPS – an academic track towards a PhD, and, on a more universal level, CHAMPS training on methods and procedures encompasses the hospital staff and laboratory technicians, demography supervisors and field workers.

## Socio-behavioral sciences research of CISM

The CISM was among the pioneering African Research Centers conducting Social Behavioral Science (SBS) research, having started in 2004 alongside a clinical trial on intermittent preventive treatment in infants (IPTi) in which recruitment was hindered by rumors regarding the administration of preventive malaria tablets spread in the community ^46^. The focus of the SBS team over the last 15 years has been to understand people's perceptions about health and disease (causes, prevention and treatment) and assess the feasibility, acceptability and adherence to new public health interventions to guide the implementation of studies and future interventions. This approach has produced strong evidence aligning with previous studies that had held that understanding cultural norms and practices is an essential step to assess the acceptability, practicality, feasibility and sustainability of interventions, which in turn informed the activities to investigate causes of death ^7, 45, 47^.

Before implementing a study, the SBS team conducts formative research to address the abovementioned intentions to inform the study and, in addition, community engagement is conducted to inform the best approaches for a successful activity. One innovation of CHAMPS was implementing a series of community entry and engagement activities based on interactive and participatory workshops with community leaders and community members at large. These workshops, entitled Participatory Inquiry Into Community Knowledge of Child Health and Mortality Prevention (PICK-CHAMP), adopted an approach to community involvement known as participatory rural assessment ^47, 48^, in which aspects related to infant mortality were discussed and debated, combined with CHAMPS intention to carry out surveillance of causes of death in children and stillbirths using MITS. Subsequently, two phases of formative research and community engagement activities were implemented to address concerns related to the feasibility and acceptability of implementing CHAMPS ^45^. Also, under the CHAMPS umbrella, rumor surveillance was instituted in the Manhiça district to anticipate possible rumors or misinformation regarding the conduct of MITS and other research studies conducted at CISM. This activity was based on establishing an active daily community information gathering system, in which CHAMPS fieldwork team and some key informants from the communities reported back to the CHAMPS community engagement team any (mis)information shared and spread among community members. The (mis)information assessed and classified as a rumor deserved a thorough follow-up and resolution from within the community and with the involvement of all key actors in the community. This approach was replicated to other CHAMPS sites

## Laboratory capacities of CISM

The CISM laboratories were established in 1997 with the aim of supporting clinical studies conducted at CISM. Since then, they have been working under international guidelines for clinical research such as Good Clinical Practice and Good Clinical Laboratory Practice (GCP and GCLP) and quality assurance with certification ISO 9001:2008 and, later on, accreditation ISO15189. CISM laboratory has a Quality Management System (QMS) with quality control allowing for regular participation in and external quality assessment for key procedures in all areas supported by external providers.

Currently, the CISM laboratory is fully equipped with the latest technology, including capacity in parasitology, haematology and biochemistry, immunology, microbiology, mycobacteriology (level III laboratory) and molecular biology. CISM Scientists and laboratory staff support morbidity surveillance and study-specific protocols, allowing detection and identification of infectious organisms using standard procedures and monitoring the communities' health, focusing on pathogens of interest ^30, 35, 38^.

Extensive work on the molecular epidemiology of different pathogens in the molecular biology laboratory has contributed to describing new genotypes/serotypes or subclones ^49^. Additionally, CISM mycobacteriology laboratory was one of the pioneers in assessing the burden of tuberculosis in post-mortem specimens using an in-house real-time polymerase chain reaction (PCR) and the Xpert MTB/RIF Ultra (Xpert Ultra) assay, reinforcing the available capacities ^50, 51^.

While working with a wide range of samples, CISM implemented a Laboratory Information Management System (LIMS) software, which uses a unique identifier (barcode label) for sample management and archiving. This system has allowed storage of all related data in centralized databases and management of the long-lasting biobank obtained throughout decades of clinical research and surveillance studies.

Under the CHAMPS grant, the laboratory capacities have been improved by introducing the CHAMPS-customized TaqMan Array Cards ^52^ – a quantitative PCR-based approach – for screening and detecting a wide range of pathogens of high public health interest. Furthermore, the Pathology Laboratory of the MCH has benefited from the purchase of a slide scanner, offering an opportunity for telepathology sessions between Mozambican pathologists and other experts globally as part of diagnostic improvement.

## The information technology, data management and analysis capacities

Prior to CHAMPS, CISM already had an extensive and well-structured data management department that supported all studies conducted at CISM and offered centralized data management to multicenter studies. The The Information Technology and Data Management and Analysis department (TIGA) department guarantees the information technology services, data management and data analysis, which comprises: a) an information technology unit, which provides a network infrastructure (wired and wireless) and servers on top of which all the electronic systems run and communicate; b) a Data Management and Statistical Analysis unit, whose tasks include database and electronic form design, data cleaning, data visualization, data analysis and production of statistical reports; and c) a data center unit that performs data entry and storage and paper reception and archiving for paper-based data collection.

## Inter-Institutional Relations, Advocacy and Communications Unit

The communication strategy of CHAMPS falls within the specific activities of the Inter-Institutional Relations, Advocacy and Communication Unit (URIAC) of CISM. Established at the end of 2014, this unit aims mainly to improve the ability to fund-raise and strengthen institutional relationships and communication. In the context of CHAMPS, the URIAC’s main goal is to ensure good visibility and image of the program and four other specific objectives: i) ensuring an adequate flow of information sharing between the communication teams of the different sites; ii) ensuring the flow of information sharing and definition of messages on mortality within the scope of the National Health Observatory in Mozambique (Observatório Nacional de Saúde - ONS); iii) disseminating data to inform public health policies and actions in real-time (through the Emory University dashboard). At the national level, CHAMPS shares data with the National Directorate of Public Health at the Ministry of Health, where all the Public Health Programs (HIV, Malaria, Tuberculosis, and tropical neglected diseases) are represented. The data is also shared with the INS Mortality Platform, which includes Health Partners (governmental and non-governmental). In addition, infographics and policy briefs (HIV and prematurity) are produced to inform public authorities to contribute to the development of concrete strategies to reduce child mortality in the country; and iv) ensure scientific dissemination and visibility of activities. Through CHAMPS, CISM has established contact with the communication team of the National Health Institute, created a database of journalists from the main media in Manhiça and the province of Zambézia (where the second CHAMPS site is now running), and improved the capacity of journalists and members of the communication team to disseminate activities on topics related to ongoing studies on causes of death

## Early learnings from the site

Despite the many challenges posed by a project of this nature, CISM started CHAMPS activities respecting local beliefs and restrictions regarding the handling and managing of deceased bodies. Moreover, the expansion of MITS to the community level in March 2019, and the call of community leaders to proactively report community deaths, among others, brought a different perspective to the work of the entire CISM investigator team. Also, establishing a rumor surveillance strategy, led by the CHAMPS SBS team from CISM (and shared with other network sites), facilitated an understanding of the need to set up an emergency community-based transport system for critically ill patients after triage by community health workers. This transport system created to address one of the community's most important priorities represents a big step towards improved health for community members and encourages community acceptance of CHAMPS and MITS in this rural and poor setting and will likely help to reduce deaths related to poor access to the health care system.

Additionally, CHAMPS data were critical to identifying certain potential actionable "hot issues" to reduce child mortality at different levels, as summarized in Table 2.

**Table 2.** CHAMPS selected actionable “hot issues” to reduce child mortality in the area at different levels of intervention.

Level	Proposed activities in response to CHAMPS DeCoDe findings on the preventability of deaths
National level	Share preliminary results and recommendations to prevent future deaths
	Quick diagnosis for level 3 notifiable conditions to allow an agile response from the decision-makers
	Improve data quality by comparing the cause of death between the Hospital and DeCoDe to allow corrections in data collected through the DHS2 *
Provincial level	Identify critical topics to train staff of the Ministry of Health (MOH) for better management of childhood diseases
District level	Share CHAMPS results with Manhiça health facilities and local government to guide new health policies
	Promote health fairs to raise awareness about women and child health
Health facility level	Train MOH staff for proper completion of medical records and death certificates
	Improve local capacity to prevent and control nosocomial infections
	Support the health facilities in training and equipment for immediate obstetric and newborn health care
	Improve antenatal care training for the medical staff to better identify and manage pregnancies with high obstetric risk
Community and family level	Radio dissemination of key messages to reduce child mortality
	Set up a community-based transport system for critically ill patients
	Share cause of death results with community leaders and families for behavior change
	Improve education for health and environmental sanitation
	Conduct family feedback with cause of death results and link families to a health facility for further care and treatment

**Legend**: DeCoDe=panel of experts to discuss and attribute the cause of death; DHS2= Mozambican MOH software used to introduce health regarding health.

* DHIS2= District Health Information Software 2

Data from CHAMPS now provide continuous monitoring of policy progress and resource allocation in Mozambique. Additionally, CHAMPS data are fed to the Mortality Platform in the ONS of Mozambique, where the CoD results are categorized in three different layers ( Table 3) to ensure that the information is reviewed, prioritized and acted upon according to its urgency and potential to enhance child survival.

**Table 3.** Categorization of the cause of death results according to public health relevance, as fed into the Mortality Platform at the National Health Observatory of Mozambique.

Category level	Description
Category 1	Common diseases in children under 5 years in Mozambique which do not require an expedited review by the Mortality Platform before the data are released. Example: malaria deaths
Category 2	Rare/unexpected diseases in children under 5 years in Mozambique that are not public health emergencies. These must be reviewed by the Mortality Platform before the data are released. Example: dengue-associated deaths.
Category 3	Public Health emergencies. These must be reported immediately to the National Directorate of Public Health [Direcção Nacional de Saúde Pública (DNSP)] and to the National Institute of Health [Instituto Nacional de Saúde (INS)] through the Mortality Platform. Example: an Ebola hemorrhagic fever-related death.

Ethical clearance for the HDSS data collection (demographic and morbidity platforms) by Manhiça HDSS was obtained both from the Institutional Bioethics Committee for Health of CISM as well as from National Bioethics Committee for Health of Mozambique. Informed consent is obtained to enroll every member of the HDSS. Verbal consent is sought during follow-up visits or update rounds, and additional informed consent is required for verbal autopsy data collection. For each specific study, despite participants having consented to be part of the HDSS, study-specific informed consent is required to recruit participants. Depending on the type of study, protocols that do not involve vulnerable participants, the collection of biological samples, or invasive procedures are reviewed by the Institutional Bioethics Committee. For more complex studies, protocols are first reviewed by the Institutional Ethics Committee and then submitted to the National Bioethics Committee for Health for further review. The CHAMPS research protocol was approved by the National Bioethics Committee for Health of Mozambique (Ref:285/CNBS/16) and adhered to the ethical standards set forth by the Declaration of Helsinki. Informed is obtained from all participants or their legal guardians before their enrollment in the CHAMPS study. For minors, consent was provided by their parents or legal guardians. Participation is entirely voluntary, and guardians are informed that their personal information will be kept confidential and used exclusively for research purposes within the scope of the CHAMPS study

## Conclusions

The CHAMPS activities have brought significant improvements to CISM's mortality surveillance by implementing advanced diagnostic techniques, better death notification and data management, and enhanced training for healthcare workers and community members, especially mothers and community leaders. This has led to more accurate and comprehensive tracking of mortality causes. The project results have unveiled numerous possibilities for designing evidence-based policies to improve child survival. This includes targeted interventions based on identified risk factors, which can inform public health strategies and tailor healthcare services to local needs. The high-quality data generated by CHAMPS have created opportunities for collaboration with health organizations, innovative research, and real-time monitoring of health interventions. The main challenge is to effectively use this data to drive impactful public health changes, such as developing robust data-sharing platforms and engaging policymakers with actionable insights. Due to the noted decline in child mortality in Manhiça, CHAMPS activities have expanded to Quelimane, where child mortality rates are the highest in the country. This expansion aims to replicate successful strategies from Manhiça leveraging the transfer of expertise and capacity. Findings from Quelimane, combined with lessons from Manhiça, will contribute to a more precise picture of the main causes of child death in Mozambique and the best strategies to address the preventable causes of mortality. This comprehensive approach will help craft tailored public health policies, ultimately improving child health outcomes across the country.

## Data Availability

No underlying data are associated with this article. Raw and primary data collected within the scope of the CHAMPS project in Mozambique are stored on CISM servers as the primary owner. For access and use, these must comply with the institutional and ethical requirements of the country, subject to CHAMPS approval. Summarized and aggregate data from the project are publicly available through the CHAMPS website: https://champshealth.org/data/. Requests for further detailed data, for research and evaluation purposes, can be made through https://champs.emory.edu/redcap/surveys/?s=PCEERX993Y.
