# Supplementary material for: Child Health and Mortality Prevention Surveillance (CHAMPS): Manhiça site description, Mozambique
Source: Gates Open Res. 2024 Sep 13;7:4. Originally published 2023 Jan 20. [Version 3] doi: 10.12688/gatesopenres.13931.3 (PMC11374382; doi:10.12688/gatesopenres.13931.3)
Supplement: Peer review history

# Reviewer report: Reviewer response for version 1

**DOI:** 10.21956/gatesopenres.15225.r33048

**Published:** 2023-04-25

**Authors:** Sanjay Juvekar

**Affiliations:** 1 Vadu Rural Health Program, KEM Hospital Research Centre, Pune, Maharashtra, Ireland; 2 Independent Consultant, Pune, Maharashtra, India

At the outset, let me say that the paper is very nicely written and there is good clarity in every section. However, there are some edits that will make this paper even more lucid for people to understand.

1. Page 03 of 22: Why CHAMPS in Mozambique? The previous sections describe how good the CISM system is in place especially the HDSS. it contradicts the justification but if in addition, if you are able to give limitations of HDSS in getting mortality information in its totality, it will support the need of CHAMPS in your area.  
2. As reviewer one has brought up all grammar & composition mistakes, I agree with all of those and I am not repeating except one on Page 9 of 22, a sub title reads as "The OF information technology......". The authors need to complete it appropriately. 
3. Page 9 of 22: there is a mention of ".....rumor surveillance was instituted in the CHAMPS umbrella". Two points here. One - it will be better if you clarify as to how exactly was the rumour surveillance done and at what frequency. Second, you mention it was instituted - do you mean to say it was initiated for this particular project and you want to make it an institutional activity which obviously makes more sense and only in the latter case the word instituted makes more sense. Otherwise one could simply say it was initiated.
4. Page 9 of 22: In section Inter Institutional, Advocacy & Communication Unit the point iii) mentions Disseminating data to inform public health policies and actions in real time. This is ambiguous, it needs explanation and maybe citing an example to support the statement. 
5. Page 10 of 22: In the conclusions there are three places where more clear explanation is needed or need elaboration. This is necessary as all these three statements appear to be abstract if not supported with explanation. It includes:
  5a) Positive changes were triggered by CHAMPS in CISM mortality surveillance.
  5b) Myriad of possibilities.
  5c) Many exciting opportunities have arisen.
6. Data Availability: The data availability is very clear but when we click the link to access data, it is confusing because it does not inform who owns the data. I am very concerned about this; making data available publicly is absolutely fine in the global interest but the ownership of these data must be very clearly mentioned which it doesn't. It gives a sense as if CHAMPS is the owner of the data which it is not in reality. In this case, Mozambique as a country owns the data and data availability must clearly mention that the data ownership is with the site and the country and that the data will be made available on CHAMPS data portal etc. As a reviewer of the manuscript, I am aware that I have no right to comment on CHAMPS portal and hence I am only saying that data ownership and custodianship needs to be clearly mentioned in this section. 
7. Ethical considerations: Even though the ethical considerations might be explained in papers published elsewhere, it is important that even this paper describes ethical considerations and how ethics was addressed all through the study duration. It will be even better if the authors could give the changes in data collection methods, ethics training etc. that were needed especially because of the differences in HDSS data collection and adding CHAMPS to the data collections.

Does the article adequately reference differing views and opinions?

Yes

Are all factual statements correct, and are statements and arguments made adequately supported by citations?

Yes

Is the Open Letter written in accessible language?

Yes

Where applicable, are recommendations and next steps explained clearly for others to follow?

Yes

Is the rationale for the Open Letter provided in sufficient detail?

Yes

Reviewer Expertise:

Public health research, data sharing, HDSS, community based vaccine trials, burden studies.

I confirm that I have read this submission and believe that I have an appropriate level of expertise to confirm that it is of an acceptable scientific standard.

# Response

**Published:** 2024-09-03

**Authors:** Charfudin Sacoor

**Affiliations:** Centro de Investigacao em Saude da Manhica, Mozambique

Thank you for your positive preliminary appraisal of the manuscript and for providing the feedback. We have carefully considered their comments. Below, you will find our detailed, point-by-point responses to each issue raised by Reviewer #2.

**Page 03 of 22: Why CHAMPS in Mozambique? The previous sections describe how good the CISM system is in place especially the HDSS. It contradicts the justification but if in addition, if you are able to give limitations of HDSS in getting mortality information in its totality, it will support the need of CHAMPS in your area**.  

**Answer:** *We appreciate the reviewer’s comments regarding the why CHAMPS in Mozambique, and we would like to clarify with the statement below that should be updated in the text:*

*Despite the robust infrastructure and valuable mortality data provided by the Manhiça HDSS, there are limitations such as recall bias in verbal autopsies, variability in interpretations, and incomplete death certificates, particularly for home deaths. These constraints highlight the need for CHAMPS, which enhances mortality surveillance through innovative post-mortem techniques like minimally invasive tissue sampling (MITS). This approach improves accuracy and cultural acceptability, providing a more comprehensive understanding of child mortality and supporting targeted interventions to improve child survival in Manhiça.*

**As reviewer one has brought up all grammar & composition mistakes, I agree with all of those and I am not repeating except one on Page 9 of 22, a subtitle reads as "The OF information technology......". The authors need to complete it appropriately.**

**Answer:** *We appreciate the reviewer’s revision suggested above. Thank you!*

*The updated sentence will be: Information Technology, Data Management, and Analysis Capacities*

****

**Page 9 of 22: there is a mention of ".....rumor surveillance was instituted in the CHAMPS umbrella". Two points here. One - it will be better if you clarify as to how exactly was the rumor surveillance done and at what frequency. Second, you mention it was instituted - do you mean to say it was initiated for this particular project and you want to make it an institutional activity which obviously makes more sense and only in the latter case the word instituted makes more sense. Otherwise one could simply say it was initiated.**

**Answer:** *Firstly, I appreciate the reviewer's comments and suggestions. Below, I provide an updated statement providing more details about rumor surveillance as well as clarification about the term "instituted" on the mentioned points:*

1. ***Rumor Surveillance Implementation and Frequency. Here we are providing the updated statement: Also, under the CHAMPS umbrella, rumor surveillance was instituted in the Manhiça district to anticipate possible rumors or misinformation regarding the conduct of MITS and other research studies conducted at CISM. This activity was based on establishing an active daily community information gathering system, in which CHAMPS fieldwork team and some key informants from the communities reported back to the CHAMPS community engagement team any (mis)information shared and spread among community members. The (mis)information assessed and classified as a rumor deserved a thorough follow-up and resolution from within the community and with the involvement of all key actors in the community. This approach was replicated to other CHAMPS sites***

 

1. ***Institutionalization of Rumor Surveillance:*** *The term "instituted" implies that this practice was not only initiated for the CHAMPS project but was also intended to be established as a continuous, institutional activity within CISM. The successful implementation in Manhiça served as a model and was subsequently replicated at places in Mozambique where CISM is working and among other CHAMPS sites, highlighting its importance and effectiveness as an ongoing strategy to maintain community trust and support.*

**Page 9 of 22: In section Inter Institutional, Advocacy & Communication Unit the point iii) mentions Disseminating data to inform public health policies and actions in real time. This is ambiguous, it needs explanation and maybe citing an example to support the statement.**

****

**Answer:** *We appreciate the reviewer’s comments and suggestion. Thank you!*

*The updated statement will be:*

*The communication strategy of CHAMPS falls within the specific activities of the Inter-Institutional Relations, Advocacy and Communication Unit (URIAC) of CISM. Established at the end of 2014, this unit aims mainly to improve the ability to fund-raise and strengthen institutional relationships and communication. In the context of CHAMPS, the URIAC’s main goal is to ensure good visibility and image of the program and four other specific objectives: i) ensuring an adequate flow of information sharing between the communication teams of the different sites; ii) ensuring the flow of information sharing and definition of messages on mortality within the scope of the National Health Observatory in Mozambique (Observatório Nacional de Saúde - ONS); ii) disseminating data to inform public health policies and actions in real-time* *(through the Emory University dashboard). At the national level, CHAMPS shares data with the National Directorate of Public Health at the Ministry of Health, where all the Public Health Programs (HIV, Malaria, Tuberculosis, and tropical neglected diseases) are represented. The data is also shared with the INS Mortality Platform, which includes Health Partners (governmental and non-governmental). In addition, infographics and policy briefs (HIV and prematurity) are produced to inform public authorities to contribute to the development of concrete strategies to reduce child mortality in the country; and iv) ensure scientific dissemination and visibility of activities. Through CHAMPS, CISM has established contact with the communication team of the National Health Institute, created a database of journalists from the main media in Manhiça and the province of Zambézia (where the second CHAMPS site is now running), and improved the capacity of journalists and members of the communication team to disseminate activities on topics related to ongoing studies on causes of death.*

**Page 10 of 22: In the conclusions there are three places where more clear explanation is needed or need elaboration. This is necessary as all these three statements appear to be abstract if not supported with explanation. It includes:**

**5a) Positive changes were triggered by CHAMPS in CISM mortality surveillance.**

**5b) Myriad of possibilities.**

**5c) Many exciting opportunities have arisen.**

****

**Answer:** *We appreciate the reviewer’s comments and suggestion. Thank you!*

*The conclusion statement will be:*

*The CHAMPS activities have brought significant improvements to CISM's mortality surveillance by implementing advanced diagnostic techniques, better death notification and data management, and enhanced training for healthcare workers and community members, especially mothers and community leaders. This has led to more accurate and comprehensive tracking of mortality causes. The project results have unveiled numerous possibilities for designing evidence-based policies to improve child survival. This includes targeted interventions based on identified risk factors, which can inform public health strategies and tailor healthcare services to local needs. The high-quality data generated by CHAMPS have created opportunities for collaboration with health organizations, innovative research, and real-time monitoring of health interventions. The main challenge is to effectively use this data to drive impactful public health changes, such as developing robust data-sharing platforms and engaging policymakers with actionable insights. Due to the noted decline in child mortality in Manhiça, CHAMPS activities have expanded to Quelimane, where child mortality rates are the highest in the country. This expansion aims to replicate successful strategies from Manhiça, leveraging the transfer of expertise and capacity. Findings from Quelimane, combined with lessons from Manhiça, will provide a clearer picture of the main causes of child death in Mozambique and the best strategies to address preventable mortality. This comprehensive approach will help craft tailored public health policies, ultimately improving child health outcomes across the country.*

*.* **Data Availability: The data availability is very clear but when we click the link to access data, it is confusing because it does not inform who owns the data. I am very concerned about this; making data available publicly is absolutely fine in the global interest but the ownership of these data must be very clearly mentioned which it doesn't. It gives a sense as if CHAMPS is the owner of the data which it is not in reality. In this case, Mozambique as a country owns the data and data availability must clearly mention that the data ownership is with the site and the country and that the data will be made available on CHAMPS data portal etc. As a reviewer of the manuscript, I am aware that I have no right to comment on CHAMPS portal and hence I am only saying that data ownership and custodianship needs to be clearly mentioned in this section**.

**Answer:** *We appreciate the reviewer’s comments on this issue. Thank you!*

*An additional sentence was added to the statement to clarify the location and ownership of primary data and how this can be accessed. Now the statement will be:*

*Raw and primary data collected within the scope of the CHAMPS project in Mozambique are stored on CISM servers as the primary owner. For access and use, these must comply with the institutional and ethical requirements of the country, subject to CHAMPS approval. Summarized and aggregate data from the project are publicly available through the CHAMPS website:* https://champshealth.org/data/. *Requests for further detailed data, for research and evaluation purposes, can be made through* https://champs.emory.edu/redcap/surveys/?s=PCEERX993Y

**

**Ethical considerations: Even though the ethical considerations might be explained in papers published elsewhere, it is important that even this paper describes ethical considerations and how ethics was addressed all through the study duration. It will be even better if the authors could give the changes in data collection methods, ethics training etc. that were needed especially because of the differences in HDSS data collection and adding CHAMPS to the data collections.**

**Answer:** *We appreciate the reviewer’s comments and suggestions. Thank you!*

*A specific ethical consideration statement was added to explain the procedures in place regarding CHAMPS activities:*

*Ethical clearance for the HDSS data collection (demographic and morbidity platforms) by Manhiça HDSS was obtained both from the Institutional Bioethics Committee for Health of CISM as well as from National Bioethics Committee for Health of Mozambique. Informed consent is obtained to enroll every member of the HDSS. Verbal consent is sought during follow-up visits or update rounds, and additional informed consent is required for verbal autopsy data collection. For each specific study, despite participants having consented to be part of the HDSS, study-specific informed consent is required to recruit participants. Depending on the type of study, protocols that do not involve vulnerable participants, the collection of biological samples, or invasive procedures are reviewed by the Institutional Bioethics Committee. For more complex studies, protocols are first reviewed by the Institutional Ethics Committee and then submitted to the National Bioethics Committee for Health for further review. The CHAMPS research protocol was approved by the National Bioethics Committee for Health of Mozambique (Ref:285/CNBS/16) and adhered to the ethical standards set forth by the Declaration of Helsinki. Informed is obtained from all participants or their legal guardians before their enrollment in the CHAMPS study. For minors, consent was provided by their parents or legal guardians. Participation is entirely voluntary, and guardians are informed that their personal information will be kept confidential and used exclusively for research purposes within the scope of the CHAMPS study*

 Again we thank reviewer #1 and reviewer #2 for their comments that will help to improve the manuscript. As per your instructions, all changes made to the main body of the text are presented in track change in the updated version of the manuscript.

**Corresponding author**

**Charfudin Sacoor, **Manhiça Health Research Center, Municipio da Vila da Manhiça, 1929, Mozambique

# Reviewer report: Reviewer response for version 1

**DOI:** 10.21956/gatesopenres.15225.r32866

**Published:** 2023-03-03

**Authors:** Mark Collinson

**Affiliations:** 1 MRC/Wits Rural Public Health and Health Transitions Research Unit, School of Public Health, University of the Witwatersrand, Johannesburg, South Africa

This open letter provides a site description of a known and respected Health and Demographic Surveillance System (HDSS) in Mozambique, the Manhiça HDSS run by the Manhiça Health Research Center (CISM). It is a valuable contribution to knowledge because it provides a description of the research platform and the foundational methodology and purpose of the CHAMPS programme. The Manhiça HDSS in Mozambique was the first HDSS to implement the CHAMPS methods and implement the developments for improving the precision of and knowledge on child, infant and neonatal mortality.

 Overall, the manuscript contains what it needs to be published and is well written, but three things need to be addressed and several minor text edits considered.

**Issues to be addressed:**

1. A short description on the migration patterns in this context would be good.  
2. A paragraph on strengths and weaknesses of the HDSS method would be helpful.
3. There should be an explanation of how people consent to participate in the different parts of the research and how the ethics oversight process works.

**Edits recommended:**

1. Heading: edit suggestion:

- “Child Health and Mortality Prevention Surveillance (CHAMPS): Manhiça, site description, Mozambique”
  to
  “Child Health and Mortality Prevention Surveillance (CHAMPS): Manhiça, Mozambique, site description”
- Locating the document in Mozambique is good but links with the name Manhiça, so it should be:
  …Manhiça, Mozambique, …”
- and the type of document is a site description, so this must come at the end.
  … Manhiça, Mozambique, site description”

2. Page 3. Para 3. edit suggestion:

- “ … as part of the tools used…”
  to
  “ … as one of the tools used…”
  or
  “ … as part of the tool-kit used…”

3. Pg 3. Para 10 (last one) edit suggestion:

- “The district is located in a plain…”
  to
  “The district is located on a plain…”

4. Pg 3. Para 10 (last one) edit suggestion:

- “…dry season, lasting…”
  to
  “…dry season lasting…”

5. Table 1. caption edit suggestion:

- “Selected demographic and public health indicators in Mozambique and Manhiça.”
  to
  “Selected demographic and public health indicators in Mozambique and Manhiça HDSS.”
  To remind the reader that there are different methods being used to produce the data, i.e. the first column is using national census data and the second column HDSS data, and it is not just a geographical difference.

6. Table 1. Column headers edit suggestion:

- Third column header:
  “Manhiça (Year 2020)”
  to
  “Manhiça HDSS (Year 2020)”
  For the same reason given in suggestion 5 above.

7. Pg 6. first line. Typo:

- “in thecountry”
  to
  “in the country”

8. Pg 6. Second para. Middle edit suggestion:

- “When the CHAMPS network was established back in…”
  to
  “When the CHAMPS network was established in…”

9. Pg 6.  Para 5. End. typo:

- “…in the past24 hours”
  to
  “…in the past 24 hours”

10. Pg 7. Para 8. End. edit suggestion:

- “…investigate the cause of death…”
  to
  “…investigate the causes of death…”
- and
  “…recognising significant barriers in terms of acceptability that conventional post-mortem approaches may pose to communities.”
  to
  “…recognising significant barriers to acceptability that conventional post-mortem approaches may pose.”

11. Pg 8. Para 1. Help with acronyms:

- Spell out MCH when used for the first time.
- And possibly write out the full name MDH again because it is a few pages since the reader was last informed about the Manhiça District Hospital.

12. Pg 9. Para 8. Help with acronyms:

- Spell out TIGA.

13. Pg 9. Para 7. Edit suggestion:

- “…electronic forms design,…”
  to
  “...electronic form design,…”

14. Pg 9. Para 8. Edit suggestion:

- “…communication strategy on CHAMPS..”
  to
  “…communication strategy of CHAMPS..”

15. Pg 9. Para 8. Edit suggestion:

- “… improve the ability to influence, fund-raise and strengthen…”
  to
  “… improve the ability to fund-raise and strengthen…”

16. Table 2:

- Provide asterisks for the legend
- “…DeCoDe findings…”
  to
  “…*DeCoDe findings…”
  with the accompanying footnote amended.
- And adding ** to the second legend component, i.e. “…*DHS2=…”

17. Pg 11. Para 1. Last sentence:

- “…, will certainly contribute to a better picture…
  to
  “…, will contribute to a more precise picture…
- and
  “…to address those preventable causes…”
  to
  “…to address the preventable causes…”

Does the article adequately reference differing views and opinions?

Yes

Are all factual statements correct, and are statements and arguments made adequately supported by citations?

Yes

Is the Open Letter written in accessible language?

Yes

Where applicable, are recommendations and next steps explained clearly for others to follow?

Partly

Is the rationale for the Open Letter provided in sufficient detail?

Yes

Reviewer Expertise:

Population and Public Health

I confirm that I have read this submission and believe that I have an appropriate level of expertise to confirm that it is of an acceptable scientific standard.

# Response

**Published:** 2023-04-03

**Authors:** Charfudin Sacoor

**Affiliations:** Centro de Investigacao em Saude da Manhica, Mozambique

First of all, I would like to thank the reviewer for all the comments and suggestions made. Below are the answers to the points raised by him and which will require some minor edits on the current version of manuscript:

1. **A short description on the migration patterns in this context would be good**

Manhiça and most of the rural districts in southern Mozambique are the main points of origin for migrants living or in permanent exchanging with South Africa, Eswatini and the greater Maputo city region. Historically, these rural areas have provided large numbers of labour migrants to work in mines and on commercial farms in these two neighbouring countries but also to seek better living conditions in the Mozambican capital.

 In 2019, immediately before the Covid-19 pandemic, the crude external out-migration rate was 85.8 per 1000 person year’s observation (pyrs) among males and 77.7 per 1000 pyrs among females. During the pandemic period, specifically for the year 2021, the external out-migration rate became 65.3 per among males and 60.4 per 1000 pyrs among females. The out-migration phenomenon is accentuated in the young population, especially in the 15-39 age interval.

 In relation to external in-migration, considering the year before the start of the covid-19 pandemic (2019), we can see the same trend if compared with external out-migration, where males present the highest rate of in-migration if compared with the rate in females, therefore being 46.3 per 1000 pyrs males and 43.7 per 1000 pyrs for females. During 2021, in the course of the pandemic the external in-migration was 27.3 and 27.5 per 1000 for male and females respectively. An important aspect to mention, is high excess of the external in-migration among children under age of five (rate of 40-68 per 1000 pyrs during these 3 years), probably brought from outside the study area to stay with their grandparents or even accompanying their parents when their return to establish in Manhiça.

1. **A paragraph on strengths and weaknesses of the HDSS method would be helpful.**

The HDSS and the Manhiça HDSS in particular provides ongoing and periodically updated households and population counts (births, deaths, pregnancy, migration) and other relevant socio-demographic information such as households characteristics, socio-economic status of the households, etc . This platform is also important to understand how local contextual factors, environmental, demographic, and programmatic factors can affect the health outcomes. In Manhiça, children under the age of 15 years including children under 5 years who are part of CHAMPS study, their outpatient and in-patient visits to the health facilities within the HDSS are also collected. In addition, this platform allows conducting clinical trials, monitor the national and global health intervention and programmes as well as to computed accurate demographic and epidemiological indicators. Due to spatial limitation, data generated within the HDSS cannot be generalized to larger populations. Cost and respondent fatigue are also some issues to consider as weaknesses of this method.

1. **There should be an explanation of how people consent to participate in the different parts of the research and how the ethics oversight process works.**

As HDSS deals with enumerations from households and all data, collection follows institutional, national and international policies and procedures or guidelines for protecting information from human participants and maintaining confidentiality. Informed consent are provide to enrol every member of the HDSS. Verbal consent is asked during the follow-up visits or update rounds. Additional informed consent is required for verbal autopsy data collection. For each specific study, despite the participants being consented to be part of the HDSS, it will require to sign the study specific informed consent to recruit a participant. Depending on the type of studies, the protocols that do not involve vulnerable participants, the collection of biological samples or invasive studies, these are reviewed by the Institutional bioethics committee and for more complex studies, protocols are first reviewed by the institutional ethics committee and then submitted to the National Bioethics Committee for Health also to be reviewed by this committee.

**Edits recommended:**

1. Heading: edit suggestion:

- “Child Health and Mortality Prevention Surveillance (CHAMPS): Manhiça, site description, Mozambique”
  to
  “Child Health and Mortality Prevention Surveillance (CHAMPS): Manhiça, Mozambique, site description”
- Locating the document in Mozambique is good but links with the name Manhiça, so it should be:
  …Manhiça, Mozambique, …”
- and the type of document is a site description, so this must come at the end.
  … Manhiça, Mozambique, site description”

**1. Answer: **We appreciate the reviewer’s edits suggested above. Thank you!.

 The updated heading will be: “Child Health and Mortality Prevention Surveillance (CHAMPS): Manhiça, Mozambique, site description”

2. Page 3. Para 3. edit suggestion:

- “ … as part of the tools used…”
  to
  “ … as one of the tools used…”
  or
  “ … as part of the tool-kit used…”

**2. Answer: **We appreciate the reviewer’s edits suggested above. Thank you!

 The updated sentence will be: *T* *he sharp innovation brought by CHAMPS in Manhiça was the inclusion, as a routine activity, of the minimally invasive tissue sampling technique [MITS, also known as Minimally Invasive Autopsy (MIA)] **as part of the tool-kit** used to investigate causes of death (CoD) in stillbirths and children under the age of 5 years* *[2, 3]*

3. Pg 3. Para 10 (last one) edit suggestion:

- “The district is located in a plain…”
  to
  “The district is located on a plain…”

**3. Answer: **We appreciate the reviewer’s edits suggested above. Thank you!

 The updated sentence will be: *The **district is located on a plain**, surrounded by the Incomati River, covering an area of 2.380km² This district has two distinct climate seasons.*

4. Pg 3. Para 10 (last one) edit suggestion:

- “…dry season, lasting…”
  to
  “…dry season lasting…”

**4. Answer: **We appreciate the reviewer’s edits suggested above. Thank you!

 The updated sentence will be: *The warm season (November to April) coincides with most of the rainfall (900 - 1100 mm on average) and is followed by a cool and dry season lasting for the rest of the year.*

5. Table 1. caption edit suggestion:

- “Selected demographic and public health indicators in Mozambique and Manhiça.”
  to
  “Selected demographic and public health indicators in Mozambique and Manhiça HDSS.”
  To remind the reader that there are different methods being used to produce the data, i.e. the first column is using national census data and the second column HDSS data, and it is not just a geographical difference.

**5. Answer: **We appreciate the reviewer’s edits suggested above. Thank you!

 The updated sentence will be: *Table 1. Selected demographic and **public** health indicators in Mozambique and Manhiça **HDSS.*** 

6. Table 1. Column headers edit suggestion:

- Third column header:
  “Manhiça (Year 2020)”
  to
  “Manhiça HDSS (Year 2020)”
  For the same reason given in suggestion 5 above.

**6. Answer: **We appreciate the reviewer’s edits suggested above. Thank you!

*The updated header in the third column of  table 1 will be:* **Manhiça HDSS (year 2020)**

7. Pg 6. first line. Typo:

- “in thecountry”
  to
  “in the country”

**7. Answer: **We appreciate the reviewer’s edits suggested above. Thank you!

*The updated sentence will be: Over the past 25 years, the CISM has conducted a series of studies with an important impact on public health policies **in the country**, including studies on malaria preventive tools (RTS,S), a malaria candidate vaccine* *[23]* *, intermittent preventive treatment in infants and intermittent preventive treatment in pregnancy (IPTi/IPTp)* *[24, 25]* *), the treatment of malaria* *[26, 27]* *, and the detailed description of the burden and epidemiology of malaria, childhood bacterial infections (including pneumonia or meningitis), moderate-to-severe diarrhea and other life-threatening infections in children* *ADDIN EN.CITE.DATA* *[28-33]* *.*

8. Pg 6. Second para. Middle edit suggestion:

- “When the CHAMPS network was established back in…”
  to
  “When the CHAMPS network was established in…”

**8. Answer: **We appreciate the reviewer’s edits suggested above. Thank you!

*The updated sentence will be:* *When the CHAMPS network was established in 2014, and Manhiça applied as a potential member, the under 5 mortality rate in the area was 58.5/1000 live* births.

9. Pg 6.  Para 5. End. typo:

- “…in the past24 hours”
  to
  “…in the past 24 hours”

**9. Answer: **We appreciate the reviewer’s edits suggested above. Thank you!

*The updated sentence will be: Briefly, it includes three three types of visits, namely (i) yearly rounds that were routinely increased to every six months as a result of CHAMPS; (ii) weekly visits to key informants (about 170); and (iii) daily visits to the health facilities to gather information on pregnancies in prenatal consultations as well as deaths and pregnancy outcomes that have occurred **in the past 24 hours.***

10. Pg 7. Para 8. End. edit suggestion:

- “…investigate the cause of death…”
  to
  “…investigate the causes of death…”
- and
  “…recognising significant barriers in terms of acceptability that conventional post-mortem approaches may pose to communities.”
  to
  “…recognising significant barriers to acceptability that conventional post-mortem approaches may pose.”

**10. Answer: **We appreciate the reviewer’s edits suggested above. Thank you!

 The updated sentence will be: *Such results highlighted the need for more robust approaches to investigate the **causes** of death, recognizing the significant barriers to acceptability that conventional post-mortem approaches may pose to communities.*

11. Pg 8. Para 1. Help with acronyms:

- Spell out MCH when used for the first time.
- And possibly write out the full name MDH again because it is a few pages since the reader was last informed about the Manhiça District Hospital.

**11. Answer: **We appreciate for this comment. Thank you!

 The updated sentence will be: *The validation studies, also conducted in **Maputo Central Hospital** (MCH).*

12. Pg 9. Para 8. Help with acronyms:

- Spell out TIGA.

**12. Answer:** We appreciate for this comment. Thank you!

 The updated sentence will be: The ***Information Technology and Data Management and Analysis department (TIGA, abbreviation in Portuguese)*** *guarantees the information technology services, data management and data analysis, which comprises:*

13. Pg 9. Para 7. Edit suggestion:

- “…electronic forms design,…”
  to
  “...electronic form design,…”

**13. Answer:** We appreciate these edits. Thank you!

 The updated sentence will be: *b) a Data Management and Statistical Analysis unit, whose tasks include database and electronic **form **design, data cleaning, data visualization, data analysis and production of statistical reports;*

14. Pg 9. Para 8. Edit suggestion:

- “…communication strategy on CHAMPS..”
  to
  “…communication strategy of CHAMPS..”

**14. Answer:** We appreciate these edits. Thank you!

 The updated sentence will be: *The communication strategy **of** CHAMPS falls within the specific activities of the Inter-Institutional Relations, Advocacy and Communication Unit (URIAC) of CISM.*

15. Pg 9. Para 8. Edit suggestion:

- “… improve the ability to influence, fund-raise and strengthen…”
  to
  “… improve the ability to fund-raise and strengthen…”

**15. Answer:** We appreciate these edits. Thank you!

 The updated sentence will be: *Established at the end of 2014, this unit aims mainly to improve the **ability to fund-raise and strengthen** institutional relationships and communication.*

16. Table 2:

- Provide asterisks for the legend
- “…DeCoDe findings…”
  to
  “…*DeCoDe findings…”
  with the accompanying footnote amended.
- And adding ** to the second legend component, i.e. “…*DHS2=…”

**16. Answer:** We appreciate these comments. Thank you!

 The updated sentence will be: *Improve data quality by comparing the cause of death between the Hospital *and **Determination of Cause of Death** (DeCoDe) to allow corrections in data collected through the **District Health Information Software 2** (DHIS2)

17. Pg 11. Para 1. Last sentence:

- “…, will certainly contribute to a better picture…
  to
  “…, will contribute to a more precise picture…
- and
  “…to address those preventable causes…”
  to
  “…to address the preventable causes…”

**17. Answer:** We appreciate these comments. Thank you!

 The updated sentence will be: *This new site will surely benefit from the capacity and expertise transfer from the original CHAMPS site in Manhiça. Results from Quelimane, in addition to the lessons learned from Manhiça, **will contribute to a more precise picture** of the principal causes of child death in the country, along with the best strategies to address **the** preventable causes of mortality.*
